# Peramivir: A Novel Intravenous Neuraminidase Inhibitor for Treatment of Acute Influenza Infections

**DOI:** 10.3389/fmicb.2016.00450

**Published:** 2016-03-31

**Authors:** Malak M. Alame, Elie Massaad, Hassan Zaraket

**Affiliations:** ^1^The School of Pharmacy, Lebanese International UniversityBeirut, Lebanon; ^2^Department of Experimental Pathology, Immunology, and Microbiology, Faculty of Medicine, American University of BeirutBeirut, Lebanon; ^3^Center for Infectious Diseases Research, Faculty of Medicine, American University of BeirutBeirut, Lebanon

**Keywords:** influenza, neuraminidase inhibitors, peramivir, efficacy, safety

## Abstract

Peramivir is a novel cyclopentane neuraminidase inhibitor of influenza virus. It was approved by the Food and Drug Administration in December 2014 for treatment of acute uncomplicated influenza in patients 18 years and older. For several months prior to approval, the drug was made clinically available under Emergency Use authorization during the 2009 H1N1 influenza pandemic. Peramivir is highly effective against human influenza A and B isolates as well as emerging influenza virus strains with pandemic potential. Clinical trials demonstrated that the drug is well-tolerated in adult and pediatric populations. Adverse events are generally mild to moderate and similar in frequency to patients receiving placebo. Common side effects include gastrointestinal disorders and decreased neutrophil counts but are self-limiting. Peramivir is administered as a single-dose via the intravenous route providing a valuable therapeutic alternative for critically ill patients or those unable to tolerate other administration routes. Successful clinical trials and post-marketing data in pediatric populations in Japan support the safety and efficacy of peramivir in this population where administration of other antivirals might not be feasible.

## Introduction

Influenza virus infections cause significant morbidity and mortality during annual outbreaks and result in large economic losses due to healthcare costs and loss of productivity (Nicholson et al., 2003). These outbreaks occur during the winter season in countries with temperate weather and during rainy seasons in tropical countries (Tamerius et al., 2010). Influenza pandemics periodically occur due to the emergence of antigenically novel influenza viruses in humans and can pose a threat for higher morbidity and mortality rates than seasonal outbreaks (Salomon and Webster, 2009). In the last century four major pandemics have occurred: the Spanish influenza (1918), the Asian influenza (1957), the Hong Kong influenza (1968), and the 2009 H1N1 pandemic influenza (H1N1pdm09; Cox and Subbarao, 2000). Currently, several avian-origin influenza strains carry pandemic potential (Abdelwhab and Hafez, 2011; Tan et al., 2015). So far, influenza control efforts have focused on vaccines and antiviral therapies that target different components of the virus or host factors.

Influenza viruses belong to the *Orthomyxoviridae* family with two types, A and B, which are the cause of major outbreaks in humans. Influenza viruses have negative-sense, single-stranded, segmented RNA genomes and possess three membrane proteins, the hemagglutinin (HA), the neuraminidase (NA), and the matrix protein (M2; McGeoch et al., 1976; Palese and Schulman, 1976; Palese, 1977). The HA protein mediates sialic acid-conjugated cell receptor recognition, receptor binding, and fusion-mediated entry into cells (Sauter et al., 1992; Hamilton et al., 2012). The M2-channel (proton pump) facilitates release of viral RNA into the cytoplasm by acidifying the virus interior (de Vries et al., 2011). Virus replication commences in the nucleus and virus assembly occurs at the cell membrane (Leser and Lamb, 2005; Banerjee et al., 2013). The NA protein facilitates the release of newly assembled virus particles by cleavage of sialic acid residues (Colman, 1994). The NA also promotes virus movement through airway mucus thereby enhancing its infectivity (Yang et al., 2014).

Influenza A viruses are highly diverse with 16 HA and 9 NA subtypes identified thus far (Salomon and Webster, 2009). Wild aquatic birds are the main reservoir for influenza A viruses, which can also infect many other hosts, including humans (Webster et al., 1992). Furthermore, bats have recently been identified as the reservoir for two novel influenza-like subtypes (H17N9 and H18N10; Tong et al., 2012, 2013; Wu et al., 2014). Two influenza A subtypes (H1N1 and H3N2) and two influenza B strains (Yamagata and Victoria) are the major subtypes that cause annual outbreaks in humans (Nicholson et al., 2003). However, several avian influenza subtypes (e.g., H5N1, H7N9, and H9N2) have caused human infections and pose a pandemic threat should they adapt and acquire aerosol transmissibility among humans (Alexander et al., 2009; Abdelwhab and Hafez, 2011; Lam et al., 2015).

Influenza vaccines and antiviral drugs are effective in preventing infections or ameliorating disease severity (Osterholm et al., 2012). Live-attenuated and inactivated vaccines are available for prevention of influenza (Krammer and Palese, 2015). Two antiviral groups, M2 channel blockers and neuraminidase inhibitors (NAIs) are available for treatment and prevention of influenza infections (Jefferson et al., 2001; Stoll et al., 2003; Burnham et al., 2013). The emergence of high levels of M2 channel blocker resistance (Hata et al., 2007; Ison, 2011), have made NAIs the drugs of choice for prevention and treatment of influenza infections. Antivirals have an advantage over vaccines as they are readily available in case of the emergence of novel influenza viruses, for which the development of an effective vaccine would take several months (Wong and Webby, 2013). This was clearly demonstrated with the H1N1pdm09 emergence in 2009 during which the antiviral drug, oseltamivir, was heavily utilized while a vaccine was under development (Miller et al., 2013). Recently licensed by the Food and Drug Administration (FDA; McLaughlin et al., 2015), peramivir (BCX-1812 and RWJ-270201) is the latest addition to the already of approved NAIs, oseltamivir and zanamivir (Ison, 2011). Peramivir is administered intravenously providing an alternative access to patients unable to take medication via the oral (oseltamivir) or inhalation (zanamivir) routes. This review will focus on the development, efficacy, and safety of peramivir. *In vitro* and *in vivo* efficacies and pharmacokinetics data as well as emergence of resistance to antivirals will also be discussed.

## Design and Mode of Action of Peramivir

The active site of influenza virus NA enzyme is made up of 18 highly conserved residues (Colman, 1994). The catalytic site is made up of eight residues (R118, D151, R152, R224, E276, R292, R371, and Y406 in N2 numbering) that directly interact with the substrate (sialic acid) and participate in its catalysis. The framework site, made up of 11 residues (E119, R156, W178, S179, D/N198, I222, E227, H274, E277, N294, and E425), provides a structural framework support for the catalytic residues (Colman et al., 1983, 1993; Burmeister et al., 1992; Gubareva et al., 2001). Influenza A virus lacking the NA protein is able to infect cells but progeny viruses aggregate and fail to be released from the cell-surface (Liu et al., 1995). Similarly, blocking the enzymatic activity of the NA protein by NAIs results in the formation of virus aggregates and prevents the release of the virus from the cell surface (Palese and Compans, 1976). Elucidation of the structure and mechanism of action of the NA have made structure- and mechanism-based design of NAIs possible (Colman, 1994; Stoll et al., 2003; von Itzstein, 2007). These data have led to the identification of several potent NA inhibitors including three that have been approved for clinical use [peramivir (Babu et al., 2000), oseltamivir (Kim et al., 1997), and zanamivir (von Itzstein et al., 1993)] and one that is still under development [laninamivir (Yamashita et al., 2009), currently only approved in Japan; **Table 1**].

**Table 1:** Neuraminidase inhibitors currently approved or undergoing clinical trials for treatment and prophylaxis against influenza.

Drug	Code name	Chemical name	Brand name	Company	Route	Approval year	Reference^1^
Oseltamivir	GS4104	ethyl (3R,4R,5S)-4-acetamido-5-amino-3-pentan-3-yloxycyclohexene-1-carboxylate;phosphoric acid	Tamiflu^®^	Roche	Oral	1999	Kim et al., 1997
Zanamivir	139110-80-8	(2R,3R,4S)-3-acetamido-4-(guanidino)-2-[(1R,2R)-1,2,3-trihydroxypropyl]-3,4-dihydro-2H-pyran-6-carboxylic acid	Relenza^®^	GlaxoSmithKline	Oral inhalation	1999	von Itzstein et al., 1993
Peramivir	BCX-1812 and RWJ-270201	(1S,2S,3S,4R)-3-[(1S)-1-acetamido-2-ethylbutyl]-4-(diaminomethylideneamino)-2-hydroxycyclopentane-1-carboxylic acid	Rapivab^®^	BioCryst	Intravenous	2014	Babu et al., 2000
Laninamivir	R-125489	(2R,3R,4S)-3-acetamido-4-(guanidino)-2-[(1R,2R)-2,3-dihydroxy-1-methoxypropyl]-3,4-dihydro-2H-pyran-6-carboxylic acid	Inavir^®^	Daiichi Sankyo/Biota	Oral inhalation	-^2^	Yamashita et al., 2009

*^1^Details on nomenclature and structure can be found at https://pubchem.ncbi.nlm.nih.gov*

*^2^Laninamivir was granted approval for clinical use in Japan in 2010 and is currently used in clinical practice.*

**FIGURE 1:**
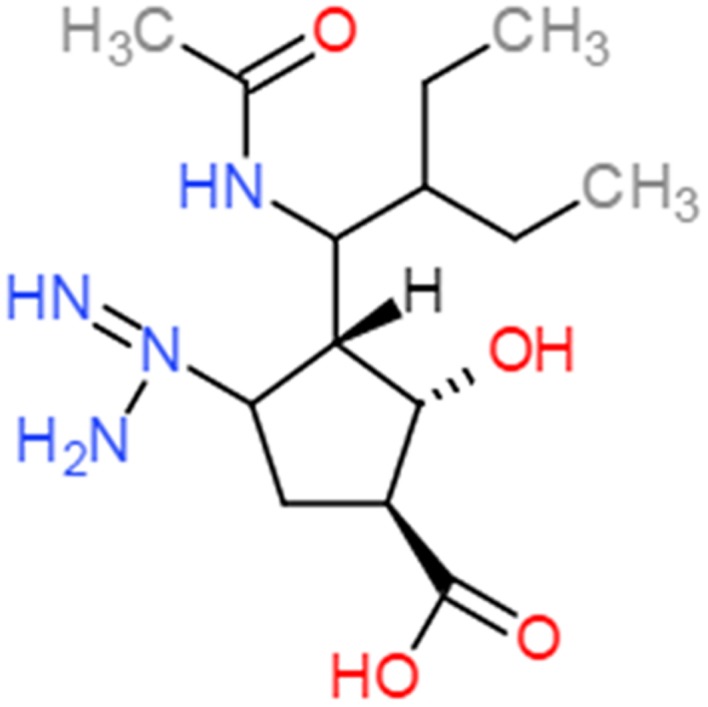
**Chemical structure of peramivir**.

**FIGURE 2:**
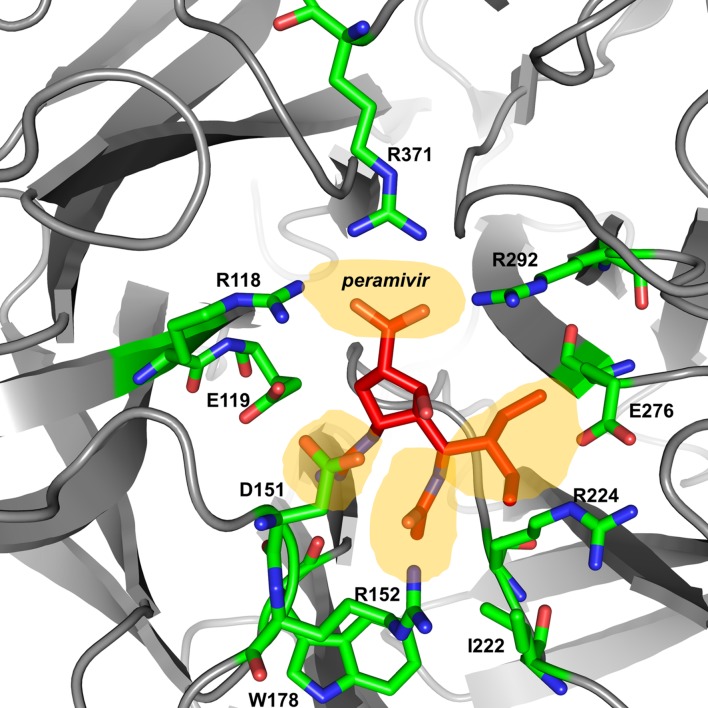
**Peramivir bound to influenza A/H7N9 neuraminidase**. Crystal structure of peramivir and influenza A/H7N9 neuraminidase complex (4 MWV). The four binding pockets of the enzyme active site are shown in yellow shades.

Peramivir was designed using crystallographic screening of enzyme-bound compounds containing a mixture of isomers to detect the most active isomer (Babu et al., 2000). It is a novel cyclopentane that is structurally distinct from other NAIs. It has a C4-guanidino substitution and a hydrophobic side chain, resembling zanamivir and oseltamivir, respectively, to take advantage of both hydrophilic pockets of the NA enzyme (**Figures 1** and **2**) (Kim et al., 1997; Babu et al., 2000; Malaisree et al., 2008). These substitutions allow tight interactions between peramivir and the enzyme active site. The negatively charged carboxylate group forms eight strong hydrogen bonds with a triad of conserved arginine residues (R118, R292, and R371) compared to only six bonds for oseltamivir and zanamivir. The methyl group of the acetamido occupies the hydrophobic pocket formed of residues W178 and I222, whereas the oxygen and nitrogen atoms form hydrogen bonds with Arg152 and a bound water molecule. The guanidino group fills the fourth pocket, forming stable hydrogen bonds and electrostatic interactions with the acidic groups of E119, D151, and E227 and replacing the existing water molecule in this pocket (Babu et al., 2000; Shetty and Peek, 2012). These interactions and the extra bonds formed between peramivir and its target, allow peramivir to tightly bind the NA active site and to have a slow dissociation-rate (*t*_1/2_ > 24 h) in comparison to oseltamivir and zanamivir (*t*_1/2_ = 1.25 h; Bantia et al., 2006).

*In vitro* activity of peramivir has been investigated against human and avian influenza A and B viruses (Babu et al., 2000; Bantia et al., 2001; Govorkova et al., 2001; Gubareva et al., 2001; Ikematsu et al., 2014, 2015a,b; Leang et al., 2014; Zaraket et al., 2014). These studies have consistently shown that peramivir possesses comparable or superior inhibitory activity against influenza A and B viruses as oseltamivir, zanamivir, and laninamivir. Influenza B isolates have been shown to be intrinsically less susceptible *in vitro* to oseltamivir compared to H1N1 and H3N2 viruses (Zaraket et al., 2010, 2014; Ikematsu et al., 2014, 2015a,b). Notably, peramivir shows improved *in vitro* activity against influenza B isolates compared to oseltamivir or zanamivir. The IC_50_ (50% inhibitory concentration) values of peramivir against influenza B were reported to be several folds lower than those of the rest of the NAIs (Dapat et al., 2010; Ikematsu et al., 2014, 2015a,b; Zaraket et al., 2014). Importantly, peramivir has been also demonstrated to be highly effective *in vitro* against emerging avian influenza viruses of pandemic potential including H5N1, H7N9, and H9N2 (Govorkova et al., 2001; Zhang et al., 2014; Marjuki et al., 2015).

## Other Influenza Virus Inhibitors

Aside from NA, at least four other viral targets have been identified for drug design based on their functions. These include the HA (Sauter et al., 1992; Mammen et al., 1995), the M2 (Hay et al., 1986), the polymerase (PB1, PB2, and PA; Hastings et al., 1996), and the NP (Tompkins et al., 2004) proteins. Furthermore, research focused on targeting host proteins and signaling pathways that play a role during influenza virus lifecycle is underway (Edinger et al., 2014).

Inhibitors targeting the HA protein can be divided into two groups based on their mode of action: (1) inhibitors *directly* targeting the HA protein, and (2) inhibitors *indirectly* targeting the HA protein (Li et al., 2015). The first group includes peptides and small molecules that interact with the HA molecule to inhibit the HA-mediated membrane fusion step (Jones et al., 2006; Zhu et al., 2011; Basu et al., 2014; White et al., 2015). It also contains molecules that bind to the HA protein disrupting its interaction with sialic acid receptors and preventing the virus from binding to the cell (Matsubara et al., 2010; Chen et al., 2015). This group also includes surfactant D and other collectins that bind to oligosaccharides on the HA molecules, hindering receptor binding (Hartshorn et al., 2008). The second group of HA inhibitors include molecules that inhibit host proteolytic enzymes (e.g., kallikrein-peptidases) necessary for cleavage-activation of the HA protein or molecules that inhibit V-ATPase dependent endosomal acidification necessary for the HA fusion-activation step (Chen et al., 2013; Leu et al., 2015).

Attempts to develop new M2-channel blockers are underway; however, these compounds seem to be inactive against mutants resistant to existing M2 inhibitors (Balgi et al., 2013). Polymerase inhibitors are highly effective against influenza virus replication (DuBois et al., 2012; Furuta et al., 2013). The most prominent drug of this group is favipiravir (T-705), a purine analog inhibitor of the viral RNA polymerase (Furuta et al., 2005; Baranovich et al., 2013). Favipiravir holds promise not only as an influenza antiviral but also against many other RNA viruses (Furuta et al., 2013). NP inhibitors include short interfering RNAs targeting conserved regions of the NP gene (Tompkins et al., 2004) or small molecules that inhibit oligomerization and subsequent nuclear import of the NP protein (Semenova et al., 2010).

In addition to drugs targeting viral proteins, several therapeutics targeting host factors are under development. Host-targeting drugs have the advantage of not selecting changes in the virus that could lead to resistance. Fludase (DAS18), a recombinant fusion protein composed of a sialidase catalytic domain derived from *Actinomyces viscosus* fused with a cell surface-anchoring sequence, cleaves sialic acid receptors from the airway epithelia, thus preventing virus attachment and infection (Malakhov et al., 2006). Nitazoxanide (NT-300), originally developed and licensed as antiprotozoal agent, is currently developed as a first-in-class broad-spectrum antiviral agent and is efficacious in reducing duration of influenza infection in Phase 2b/3 clinical trial (Haffizulla et al., 2014; Rossignol, 2014). Nitazoxanide inhibits influenza virus replication by blocking post-translational maturation of the viral HA protein (Rossignol et al., 2009). Despite the constant development of various influenza virus inhibitors, the rapid evolution of the virus makes resistance a compelling challenge.

## Drug Resistance

In 2007 and 2008, high levels of oseltamivir resistance among seasonal H1N1 viruses emerged worldwide undermining the efficacy of this drug. Nonetheless, post-pandemic 2009 influenza A and B viruses remain largely susceptible to NAIs (Ikematsu et al., 2014, 2015b; Leang et al., 2014; Meijer et al., 2014; Takashita et al., 2014; Zaraket et al., 2014). The prevalence of peramivir-resistance among influenza A/H1N1pdm09 viruses ranges between 1.3 and 3.2% and is <1% among influenza A/H3N2 and B viruses (Leang et al., 2014; Meijer et al., 2014; Takashita et al., 2014). Gubareva et al. (2001) have suggested that while resistant variants with mutations in the enzyme framework can retain susceptibility to other NAIs, those in the functional or catalytic residues tend to cause cross-resistance to all NAIs. Their study demonstrated that the most commonly detected H274Y (H275Y N1 numbering) framework mutation conferring resistance to oseltamivir results in cross-resistance to peramivir but not to zanamivir (Gubareva et al., 2001). This mutation has been shown to rapidly emerge upon intravenous (IV) treatment with peramivir in immunocompromised or hematopoietic cell transplant recipients (Memoli et al., 2010; Renaud et al., 2010). Nonetheless, in contrast to oral oseltamivir, the efficacy of peramivir against the H1N1 H274Y variant has been demonstrated *in vivo* upon single or multiple-intramuscular (IM) or IV regimens in mice, suggesting that peramivir could be used against oseltamivir-resistant H274Y influenza virus infections (Abed et al., 2011, 2012). Human influenza B isolates with H273Y mutation in the NA active site were found to be resistant to both oseltamivir and peramivir but not zanamivir (Higgins et al., 2012). Resistance to peramivir due to HA mutations has also been described *in vitro* (Smee et al., 2001). Serial passages of an H3N2 virus in the presence of peramivir caused selection of peramivir-resistant viruses linked to a K189E mutation in the HA protein.

The zanamivir-resistant influenza A/H3N2 variants with framework E119G or E119A mutations remain susceptible to peramivir and oseltamivir (Gubareva et al., 2001). Using reverse genetics, it was shown that these two mutations, as well as the E119D substitution, induce resistance to zanamivir but not to peramivir or oseltamivir in the H1N1pdm09 virus background (Baek et al., 2015). Surprisingly, laboratory generated H5N1 virus with the E119D mutation possessed high level of resistance to all NAIs. While the E119V mutation in the H3N2 context conferred resistance to oseltamivir only (Meijer et al., 2014). In influenza B, an R152K catalytic site mutation caused cross-resistance to all of the three NAIs (peramivir, oseltamivir, and zanamivir; Gubareva et al., 2001). In contrast, an H1N1pdm09 virus with this mutation remains sensitive to all three approved NAIs (Baek et al., 2015). Zanamivir-resistant influenza B R292K variant was found to be extremely resistant to oseltamivir but was moderately resistant to peramivir (Gubareva et al., 2001; Meijer et al., 2014).

Resistance to NAIs seems to arise easily among emerging influenza viruses as well. Itoh et al. (2015) reported the emergence of H7N9 influenza A virus with a R289K NA mutation conferring resistance to oseltamivir and peramivir upon NAI treatment in non-human primates. H7N9 viruses continue to cause human infections posing a pandemic threat (Zhu et al., 2016). The emergence of resistance among these viruses is a reason for concern.

Azuma et al. (2015) reported the presence of peramivir and laninamivir in high concentrations in sewage eﬄuent and river waters in Japan. This could result in passive exposure of wild birds to these NAIs and give rise to the emergence of NAI-resistance. This could be especially the case during pandemics which trigger high consumption levels of NAIs. Maintaining the efficacy of new and old antiviral drug inhibitors requires continuous monitoring of the susceptibility of circulating and emerging influenza viruses. Additionally, the ease and potential for emergence of NAI-resistant influenza viruses highlights the importance of assessing combination antiviral treatments for influenza (Govorkova and Webster, 2010). Combining antivirals targeting different influenza viral proteins and/or host molecules can hinder the emergence of resistance.

## Preclinical Studies

Numerous studies have demonstrated the efficacy of peramivir against human and emerging influenza viruses *in vivo* (Govorkova et al., 2001; Sidwell et al., 2001a; Sweet et al., 2002; Barroso et al., 2005; Bantia et al., 2006, 2011; Boltz et al., 2008; Abed et al., 2012). Earlier studies focused on assessing the oral efficacy of peramivir in comparison with oseltamivir and zanamivir treatment. Prophylactic peramivir treatment administered at a 10 mg per/kg/day by oral gavage twice daily for 5 days reduced lung viral load and provided complete protection against lethal challenge with H5N1 and H9N2 viruses in mice, similar to oseltamivir and consistent with the *in vitro* efficacies of these drugs (Govorkova et al., 2001). Similar levels of protection were demonstrated with prophylactic peramivir administration of 1–10 mg/kg/day twice daily for 5 days against H1N1, H3N2, and H6N2 viruses (Babu et al., 2000; Bantia et al., 2001; Sidwell et al., 2001a,b). Oral peramivir also significantly inhibited mortality in mice infected with influenza B virus (Sidwell et al., 2001b). Studies in ferrets suggested that 3- to 10-fold higher peramivir doses than those used in mice were needed to significantly reduce virus shedding in nasal washes and reduce inflammatory response in an H3N2 challenge model (Sweet et al., 2002). It remained unclear whether these differences were host-dependent or due to the different H3N2 strains used in each host.

Pharmacodynamics studies in mice demonstrated that the antiviral effect of peramivir was independent of the schedule used and recommended once-daily dosing as the basis for future drug evaluations (Drusano et al., 2001). Further studies showed that delayed oral administration of peramivir 48 h after H5N1 infection resulted in 50% reduced mortality in mice (Govorkova et al., 2001). In contrast, Bantia et al. (2001) reported failure of delayed treatment of H6N2 infected mice at 48 h after infection and found that full protection required initiation of treatment at 24 h after infection. Another study found that oral treatment with peramivir could be delayed at least 60 h after virus exposure and still prevent mortality in H1N1 infected mice, however, administration at 24 h after exposure provided the greatest suppression of lung virus titers (Sidwell et al., 2001b). Delayed oral peramivir treatment was also shown to be effective in immunosuppressed mice infected with H1N1 virus (Sidwell et al., 2001a). These studies emphasize the importance of accurate diagnosis and early initiation of the treatment for optimal drug efficacy.

Despite the well-established efficacy of oral peramivir in animal studies, human clinical trials resulted in less success due to low oral bioavailability (Barroso et al., 2005). Thus, further efforts were focused on exploring the parenteral route. The first study investigating the IM route in mice infected with H1N1 or H3N2 viruses demonstrated that a single, prophylactic or delayed, 10–20 mg/kg dose significantly reduced morbidity (measured by weight loss) and mortality similar to the 5-day regimen of oral peramivir (Bantia et al., 2006). This efficacy was unmatched by IM-administered oseltamivir, which failed to provide any protection. Additionally, both prophylactic and delayed treatments with a single IM peramivir injection were effective against H1N1pdm09 infection in mice (Bantia et al., 2011).

In the case of a peramivir-resistant H1N1 virus with the H274Y mutation, multiple IM doses demonstrated superior efficacy in comparison to single IM dose regimens administered as a prophylaxis in a mouse challenge model (Abed et al., 2010). IM peramivir, single and multiple doses, prevented mortality in peramivir-resistant H1N1-infected mice when it was administered at 24 or 48 h post-infection (Abed et al., 2012). Nonetheless, mice receiving peramivir IM injection at 24 h post-infection exhibited less weight loss compared to when treatment was delayed by 48 h. Post-exposure IM peramivir treatment also mitigated the disease and reduced the infectious virus titers promoting survival of mice infected with the highly pathogenic H5N1 influenza virus, although multiple doses were needed (Boltz et al., 2008; Yun et al., 2008). Contrary to the effectiveness of a single IM dose used to treat low pathogenic viruses like H1N1 (Bantia et al., 2006), a single IM injection of peramivir administered 1 h post-inoculation with a lethal H5N1 virus resulted in only 33% survival in mice (Boltz et al., 2008). Survival improved to 55% with two IM peramivir doses. An 8-day regimen consisting of two daily IM injections starting 1 h post-exposure followed by seven daily IM doses provided mice with 100% protection from lethal H5N1 challenge, but survival decreased to 78 and 56% when treatment was delayed by 24 and 48 h, respectively (Boltz et al., 2008). The 8-day regimen of 30 mg/kg/day IM peramivir, with the first dose initiated at the time of infection, also resulted in reduction of lung virus titers and mortality in mice infected with a lethal dose of the H7N9 virus (Farooqui et al., 2015).

Combination regimens of IM peramivir with oseltamivir or amantadine performed better than peramivir monotherapy against H1N1 and H3N2 viruses (Bantia et al., 2010; Smee et al., 2010). In addition, IM peramivir treatment coupled with oral favipiravir was more effective and a reduced dose was needed than in monotherapy against peramivir-resistant H1N1pdm09 virus (Park et al., 2014). Nonetheless, in the aforementioned studies, a 5-day regimen was used to test the combination therapy (Bantia et al., 2010; Smee et al., 2010; Park et al., 2014). Therefore, further research is needed to elucidate the effect of the approved single-dose treatment of peramivir in combination with other antiviral drugs.

Consistent with the effectiveness of the IM regimen, multiple studies demonstrated the efficacy of single IV injection of peramivir in animal models (Kitano et al., 2011; Kakuta et al., 2013). A single IV administration of 30 mg/kg peramivir resulted in significant reduction of nasal virus titers and clinical symptoms in ferrets and cynomolgus macaques infected with a recent influenza B virus strain, even when treatment was delayed up to 48 h post-infection (Kitano et al., 2011). IV peramivir administered as a single-dose of 30 mg/kg and 13 h after infection of mice with influenza A/H1N1 or B viruses showed significant reduction in mortality and lung viral loads, an effect that was similar to laninamivir (Kakuta et al., 2013). Immunosuppressed mice infected with the H1N1pdm09 virus required 10–20 days of daily IV peramivir to attain >80% survival, whereas a single-dose regimen was non-efficacious (Kitano et al., 2013). By contrast, repeated oral administration of oseltamivir (5 mg/kg twice daily for 20 days) failed to show any improvement in survival of infected immunosuppressed mice despite significantly reducing weight loss.

Repeated IV administration of peramivir over 5 days starting on the day of infection or 24 h after infection significantly reduced viral titers, inflammatory cytokines, and the period to virus clearance in the upper respiratory tract of cynomolgus macaques infected with H5N1 virus (Kitano et al., 2014).

Single and repeated IV peramivir administration was also shown to inhibit virus replication and proinflammatory immune responses in mice co-infected with H1N1pdm09 virus and the bacterium *Streptococcus pneumonia*, consequently leading to earlier bacterial clearance and significantly improved survival (Onishi et al., 2015; Tanaka et al., 2015). Outcomes of bacterial superinfection were significantly improved after a multiple-dose regimen of IV peramivir compared to single-dose IV peramivir or multiple oral doses of oseltamivir. Initiating treatment just after viral infection resulted in better outcomes than delaying treatment even if treatment was started before bacterial superinfection (Onishi et al., 2015). Therefore, quickly beginning peramivir administration after symptoms onset is important not only to resolve influenza virus infection, but also to prevent complications due to a subsequent bacterial infection.

Overall, animal experiments demonstrated that peramivir is as effective and in some cases more effective than other NAIs. Its safety profile in animal models and the efficacy of the single-dose regimen makes it an attractive treatment choice for influenza infections.

## Clinical Studies

### Pharmacokinetics

Experimental clinical data revealed that oral peramivir is rapidly absorbed with plasma levels beginning to rise within 1 h of administration, and the maximum concentration (C_max_) being achieved in 2.5 h (range: 2–4 h; Young et al., 2001; Barroso et al., 2005). However, oral peramivir formulation showed low bioavailability (≤3%) in humans despite the use of relatively high doses. Peak plasma levels were ∼100 ng/ml and ∼200 ng/ml after four doses of 400 and 800 mg oral peramivir, respectively (Barroso et al., 2005). This warranted the study of parenteral formulations which resulted in high drug concentrations in the blood (Boltz et al., 2008). The peak plasma concentrations for IM and IV peramivir (10,000–20,000 ng/mL) are nearly two orders of magnitude higher than those achieved with standard doses of oral oseltamivir (Hayden, 2009; Kohno et al., 2010).

The pharmacokinetics (PK) of IV peramivir were evaluated in Phase I trials in adults (Food and Drug Administration, 2015b). These trials demonstrated a linear relationship between dose and drug exposure parameters (area under the curve [AUC] and *C*_max_; Mancuso et al., 2010; Food and Drug Administration, 2015b). Plasma concentrations of IV peramivir peaked immediately after administration (Whitley et al., 2014; Yoshino et al., 2015). At the end of a 30 min IV infusion of a single-dose of 600 mg peramivir, *C*_max_ reached 46,800 ng/mL and AUC was 102,700 h × ng/mL (Food and Drug Administration, 2015b). Kohno et al. (2011a) assessed median plasma concentrations upon IV administration of single 300 or 600 mg doses over a period of 15–60 min to high-risk patients (poorly controlled diabetes, chronic respiratory tract disease, and drug-induced immunosuppression). The median plasma concentration immediately before the end of the infusion was 25,500 ng/mL in the 300 mg group and 51,500 ng/mL in the 600 mg group. Moreover, 3–9% of peramivir was detected in the nasal cavity and pharyngeal mucus of healthy subjects upon IV administration of 600 mg dose. The drug concentration in pharyngeal mucus was 5,280 nM at 2 h and 220 nM at 12 h after administration, well above its IC_50_ values (Kohno et al., 2011a). Based on a population pharmacokinetic analysis, the central volume of distribution was 12.56 L (Food and Drug Administration, 2015b).

Despite a low *in vitro* plasma protein binding of less than 30% (Chairat et al., 2013; Food and Drug Administration, 2015b), the elimination half-life of peramivir following a single IV administration of 600 mg to healthy subjects is approximately 20 h (Boltz et al., 2008; Hayden, 2009; Ison et al., 2013; Food and Drug Administration, 2015b). Together with the slow NA dissociation rate (Bantia et al., 2006), the prolonged half-life allows for infrequent dosing regimens.

Peramivir is not significantly metabolized by the liver in humans. The drug is not a substrate for CYP enzymes, does not affect glucuronidation, and is not a substrate or inhibitor of *P*-glycoprotein mediated transport (Food and Drug Administration, 2015b). Thus, dose adjustment is unnecessary in hepatic impairment (Chairat et al., 2013).

Peramivir is almost entirely eliminated by renal excretion with 90% of the drug excreted unchanged (Mancuso et al., 2010; Chairat et al., 2013; Food and Drug Administration, 2015b). Negligible accumulation was observed following multiple doses, either once or twice daily for up to 10 days (Food and Drug Administration, 2015b). Clearance of peramivir is similar to the average creatinine clearance (CrCl) for a 70 kg adult with normal renal function (Clay et al., 2011). This suggests that peramivir is excreted mainly by glomerular filtration. Moreover, CrCl was found to be the most important factor affecting peramivir PKs (Matsuo et al., 2015). Data from six clinical studies in Japan and the United States including healthy volunteers and influenza patients revealed that peramivir clearance was linearly related to CrCl when CrCl values were below 115 ml/min. On the other hand, peramivir clearance was independent of CrCl when CrCl values were over 115 ml/min (Matsuo et al., 2015). No difference in PK was observed by gender, and the effects of age and body weight on peramivir clearance were negligible (Food and Drug Administration, 2015b; Matsuo et al., 2015). Peramivir clearance was 18% higher and its distribution volume was 6% lower in influenza patients compared to healthy volunteers. However, these differences are not considered clinically significant (Matsuo et al., 2015).

Additionally, the AUC of peramivir is highly dependent on CrCl (Matsuo et al., 2015). In patients with moderate and severe renal impairment exposure to peramivir upon IV administration is expected to be increased by 3.4- and 6-fold, respectively, compared to those with normal renal function (Chairat et al., 2013). Therefore, dose adjustment based on renal function in patients with renal impairment is required to provide AUCs comparable to those in patients with normal renal function (**Table 2**) (Chairat et al., 2013; Food and Drug Administration, 2015b; Matsuo et al., 2015). Using doses as high as 600 mg IV once daily in patients with acute renal failure did not cause any toxicity suggesting a high clearance of peramivir by continuous renal replacement therapy (CRRT; Hernandez et al., 2011). Accordingly, peramivir dose should be adjusted based on the type and duration of CRRT provided. In a case report of an 18-year-old male, peramivir was well cleared by continuous venovenous hemofiltration with a half-life of 6.2 h (Scheetz et al., 2011). Peramivir was also shown to be removed by hemodialysis, with a 4-h hemodialysis reducing the systemic peramivir exposure by 73–81% (Chairat et al., 2013). In patients with chronic renal impairment maintained on hemodialysis, peramivir should be administered after dialysis at a dose adjusted based on renal function (Food and Drug Administration, 2015b).

**Table 2:** Dosage adjustment for patients with altered creatinine clearance.

Renal function	Mild renal impairment	Moderate renal impairment	Severe renal impairment
Creatinine clearance (mL/min)	≥50	30–49	10–29
Recommended dose (mg)	600	200	100

*Source: Food and Drug Administration, 2015b*.

Two studies were conducted to evaluate PK interactions between IV peramivir and other influenza antivirals (Atiee et al., 2012). Concomitant administration of peramivir with oral oseltamivir or rimantadine in humans did not adversely affect their PK profile, demonstrating a lack of drug interaction.

### Efficacy

Data from a double-blind, randomized, placebo-controlled study evaluating oral peramivir administration in experimentally infected influenza A or B patients, revealed a more pronounced drop in virus titers, regardless of virus type, in the peramivir-treated group compared to placebo (Iyer et al., 2002). These data mirrored the success of oral peramivir for treatment and prophylaxis of influenza infection in early animal studies (Bantia et al., 2001; Sidwell et al., 2001a; Sweet et al., 2002). Driven by the success of these studies, a phase III, double-blind, randomized, placebo-controlled study examined the efficacy of oral peramivir in treatment and prophylaxis of influenza A/H1N1 and B infections (Barroso et al., 2005). In the prophylaxis arm, oral peramivir was administered 24 h prior to virus inoculation and continued for a total of 5 days. Only modest, non-significant reductions in virus shedding were observed across all study arms: 58% for placebo vs. 61, 37, and 31% for influenza A with 50, 100, and 200 mg q.d. (once daily) regimens, respectively; 55% for placebo vs. 31, 45, and 47% for influenza B with 200, 400, and 800 mg q.d. regimens, respectively (Barroso et al., 2005). These values were in sharp contrast with those for prophylactic oral oseltamivir regimens of 100 mg q.d. or b.i.d. (twice daily), which completely prevented influenza A virus shedding (Hayden et al., 1999). When peramivir was administered orally 24 h after virus inoculation and continued for a total of 5 days, significant reductions in influenza A and B virus shedding rates were observed in treatment groups compared with placebo (Barroso et al., 2005). However, this effect was dose-dependent and was only observed with higher doses. The group concluded that despite peramivir tolerability, the lack of more robust antiviral effect is likely due to the relatively low oral bioavailability (<3%) compared to oseltamivir (80%) and recommended exploring alternative formulations or parenteral administration.

Intramuscular peramivir was tested in two double-blind, placebo-controlled studies in adult outpatients with acute, uncomplicated influenza (Whitley et al., 2014). In the first phase II clinical trial, 344 influenza patients were randomized to three treatment groups: single-dose peramivir 150 mg, peramivir 300 mg, and placebo. In the phase III clinical trial, 83 influenza patients were randomized to single-dose peramivir 300 mg and placebo treatment groups. The phase III study was terminated early to focus on evaluating more concentrated formulations of peramivir. In both studies, peramivir was administered within ≤36 h of symptoms onset in 66–71% of participants who were dominantly infected by H1N1 or H3N2 (Whitley et al., 2014). An integrated analysis from both studies revealed that a single-IM peramivir dose significantly shortened the time to alleviation of symptoms (113.2 h for peramivir 300 mg) compared to placebo (134.8 h); a difference of 20.7 h, adjusted *p* = 0.047. For peramivir 150 mg, the difference was 21.6 h. Similar reductions in the time to defervescence and resumption of usual activities were reported with peramivir 300 mg compared to placebo (Whitley et al., 2014). Subjects receiving 150 or 300 mg peramivir exhibited significantly lower proportions of virus shedding on days 2 and 3 and lower virus titers on day 3 compared to placebo. An improvement in the time to alleviation of symptoms in patients treated with 150 or 300 mg peramivir was associated with a significant increase in creatine kinase levels, a marker of successful IM administration (Shetty and Peek, 2012). Thus, a follow-up phase II study was conducted to evaluate to 600 mg peramivir administered as bilateral 2-mL IM injections using longer needles (BioCryst Pharmaceuticals, 2015). A total of 405 subjects with confirmed influenza infections were randomized to receive placebo or 600 mg peramivir IM. The time to alleviation of symptoms did not differ significantly between placebo (106.9 h) and 600 mg peramivir (91.1 h; *p* = 0.22). The failure of treatment was attributed to high prevalence of influenza A/H1N1 with H274Y mutation conferring resistance to peramivir among the study subjects (BioCryst Pharmaceuticals, 2015). Thus, a single-IM peramivir administration failed to overcome resistance due the H274Y mutation. Based on animal studies (Abed et al., 2011, 2012), multiple doses might be necessary to treat patients infected with resistant viruses.

Several studies investigated the effect of IV peramivir as single- or multiple-doses for treatment of influenza infection (Kohno et al., 2010, 2011b; Ison, 2011; de Jong et al., 2014). A phase II clinical trial evaluated the efficacy of single-dose IV peramivir in previously healthy adults with confirmed influenza infection (predominantly seasonal H1N1; Kohno et al., 2010). Participants (*n* = 296) were randomized to receive 300 mg peramivir, 600 mg peramivir or placebo within 48 h of symptoms onset. IV peramivir 300 and 600 mg doses significantly reduced time to alleviation of symptoms compared to placebo (300 mg 59.1 h, 600 mg 59.9 h, placebo 81.8 h; adjusted *p*-values = 0.0092 and 0.0092, respectively; Kohno et al., 2010). Defervescence was evident as early as 24 h after treatment. The proportions of subjects shedding virus were significantly smaller in the treatment groups (300 mg 36.8% and 600 mg 35.8%) compared to placebo (51.5%; *p*-values = 0.00485 and 0.0003, respectively; Kohno et al., 2010).

A multinational, multicenter, phase III study enrolled 1091 subjects in Korea, Japan, and Taiwan (Kohno et al., 2011b). The study compared single IV dose of peramivir (300 mg or 600 mg) to 5-day oral peramivir regimen (75 mg b.i.d.). Overall, the time to alleviation of symptoms was similar across all study groups: 300 mg peramivir, 78.0 h; 600 mg peramivir, 81.0 h; and oseltamivir, 81.8 h. Despite the detection of the H274Y mutation among nearly 100% of patients infected with H1N1 (Kohno et al., 2011b), the time to alleviation of symptoms in the oseltamivir group was well within the range reported in previous years when oseltamivir-susceptible H1N1 was circulating (Nicholson et al., 2000; Treanor et al., 2000). The authors concluded that the significance of the study was not compromised due to the lack of a placebo group, and that the efficacy of oseltamivir was maintained despite the prevalence of the H274Y viruses (Kohno et al., 2011b). In the H3N2-infected subgroup of patients, peramivir (300 and 600 mg doses) was as effective as oseltamivir. Surprisingly, the 300 mg but not 600 mg peramivir dose resulted in significantly shorter time to alleviation of symptoms compared to oseltamivir in the influenza B subgroup (55.3, 92.8, and 92.7 h, respectively; Kohno et al., 2011b). A smaller scale study including 32 patients hospitalized for influenza A infections in 2012 also found no significant difference in the time to defervescence between peramivir and oseltamivir groups (Yoshino et al., 2015).

Further studies assessed the efficacy of multiple doses of peramivir in severely ill or hospitalized patients. In a phase II study, Ison et al. (2013) enrolled 122 hospitalized influenza patients who were randomized to receive IV 200 or 400 mg peramivir q.d. or oral 75 mg oseltamivir b.i.d. for 5 days. The study found no significant difference in the time to clinical stability among the three treatment groups: 200 mg peramivir, 23.7 h; 400 mg peramivir, 37.0 h; and oseltamivir, 28.1 h. A *post hoc* analysis of clinical outcome of clinically unstable patients (*n* = 97) revealed similar findings (Ison et al., 2013). Consistent with earlier findings, a retrospective analysis of severely ill patients with influenza found IV peramivir to be as effective as oral oseltamivir treatment (Yoo et al., 2015). A phase III, open-label, multinational, clinical trial was conducted during the H1N1pdm09 to assess the antiviral activity of two dosing regimens of IV peramivir (300 mg b.i.d. or 600 mg q.d. for 5 days; Ison et al., 2014). The median time to resolution of symptoms was 45 h for 300 mg peramivir (*n* = 57) and 166 h for 600 mg peramivir (*n* = 70). Nonetheless, it was noted that the 600 mg group had more patients with severe disease (i.e., baseline need for ICU admission, need for supplemental oxygen, or high APACHE score), which might have attributed to the longer time to symptoms relief. No significant difference in the time-weighted change in virus titer from baseline to 48 h was observed between the treatment groups (Ison et al., 2014).

In high risk patients, defined as those with ≥1 risk factors and ≥2 moderate to severe influenza symptoms; *n* = 37), single-dose IV peramivir (300 or 600 mg) was assessed with additional administrations delivered as needed up to 5 days (Kohno et al., 2011a). The median time to alleviation of illness was significantly longer for the 300 mg peramivir (114.4 h) compared to the 600 mg dose (42.3 h). Another phase III double-blind, randomized clinical trial, assessed the efficacy of multi-dose peramivir in hospitalized patients (de Jong et al., 2014). Of 1600 subjects screened, 338 subjects with confirmed influenza (A or B) were included in the intent to treat influenza (ITTI) population. Patients were randomized to receive placebo or 600 mg IV peramivir q.d. for 5 days, in addition to the institution’s standard of care (SOC) treatment. The non-NAI SOC ITTI (did not receive concurrent NAI) population included 121 patients and the remaining 217 belonged to the NAI SOC (216 received oseltamivir and 1 received zanamivir) ITTI population (de Jong et al., 2014). The median time to clinical resolution did not differ significantly between peramivir and placebo (42.5 h vs. 49.5 h, respectively; *p* = 0.97) in the non-NAI SOC population. Similar data were observed for the NAI SOC population. An improved treatment outcome was observed in subjects enrolled within <48 h or those admitted to intensive care unit (ICU). However, the difference in time to clinical resolution between placebo and peramivir groups did not reach statistical significance due to the limited sample size and the study was terminated (de Jong et al., 2014). With the exception of this study, studies in severely ill patients are undermined by the lack of a placebo group, which is not possible in high risk populations.

Currently, IV peramivir is approved for use in children <18-year-old in Japan. The approval of peramivir in Japan was based on a multicenter, open-label, uncontrolled clinical study demonstrating efficacy and safety of IV peramivir in children (Sugaya et al., 2012). Children with confirmed influenza infection during the H1N1pdm09 and ranging in age between ≥28 days to <16 years were enrolled. Peramivir was administered as IV infusion at 10 mg/kg (600 mg maximum dose in children with ≥60 kg body weight). The median time to alleviation of symptoms was 29.1 h and virus shedding was confirmed in 78.2 and 7.1% of children on days 2 and 6 after initiation of treatment, respectively (Sugaya et al., 2012). Hikita et al. (2012) compared the efficacy of IV peramivir (10 mg/kg, max 300 mg per dose) with other NAIs. For influenza A patients, the median duration of fever was 1 day for peramivir and 2 days for zanamivir (*p* = 0.0242). In the case of influenza B, the median duration of fever was also 1 day for peramivir compared to 3 days for laninamivir (*p* = 0.0097). These results suggest that peramivir is also useful in children, especially where the use of inhalation drugs is not feasible.

### Safety

Overall, peramivir demonstrated good tolerability and safety in all clinical studies (Kohno et al., 2010, 2011b; Ison et al., 2013, 2014; Whitley et al., 2014). Post-marketing evaluation of peramivir under routine clinical settings mirrored the clinical trials in terms of safety and effectiveness in adults and pediatrics (Komeda et al., 2014, 2015). Most reported adverse events (AEs) were mild to moderate in severity and were more common in young children (Kohno et al., 2011a; Sugaya et al., 2012; de Jong et al., 2014; Ison et al., 2014; Whitley et al., 2014) and their frequency was comparable to placebo (Kohno et al., 2010; Whitley et al., 2014). Peramivir AEs were similar in severity and frequency to those reported for oral oseltamivir (Kohno et al., 2011a; Ison et al., 2013), and generally occurred within 3 days of initiating treatment and rapidly subsided (Sugaya et al., 2012). The frequency of AEs was similar regardless of the number of doses received.

The most common AEs were gastrointestinal disorders including diarrhea (mild to moderate), nausea, and vomiting (Kohno et al., 2011a; Sugaya et al., 2012; Ison et al., 2013; de Jong et al., 2014; Komeda et al., 2014, 2015; Whitley et al., 2014). Komeda et al. (2015) reported that abnormal behavior, cough, and pyrexia were among the common AEs in pediatrics along with GI disorders. The majority of serious AEs were found to be related to influenza infection (e.g., pneumonia, chronic obstructive pulmonary disorder; de Jong et al., 2014). The most common severe AEs were decrease in neutrophil count (lowest mainly on day 3) and prolonged QT interval; however, these returned to normal without any intervention (Kohno et al., 2010, 2011a; Sugaya et al., 2012; Komeda et al., 2014, 2015).

Some rare but severe AEs were also reported upon treatment with peramivir. A 44-year-old man with history of lower than normal platelet count had severe thrombocytopenia that was possibly elicited by peramivir (Harada-Shirado et al., 2014). This was confirmed by drug-induced lymphocyte-stimulating test which was positive for peramivir. Hayashi et al. (2015) described a case of exacerbated myasthenia gravis in a 73-year-old women triggered by IV peramivir.

## FDA ApprovaL

Given its well-established efficacy and safety, the FDA granted approval for IV peramivir under the brand name Rapivab^TM^ on December 19, 2014 (Food and Drug Administration, 2015b). Under the FDA approval, peramivir is indicated for treatment of acute uncomplicated influenza in patients 18 years and older who have been symptomatic for no more than 2 days. The recommended dose is 600 mg administered once via IV infusion for 15–30 min within 48 h of symptoms onset. Peramivir must be diluted prior to administration. In case of renal impairment, the recommended dose is lowered to 200 mg for patients with creatinine clearance 30–49 mL/min and to 100 mg for patients with creatinine clearance 10–29 mL/min (**Table 2**).

## Emergency Use Authorization

On October 23, 2009 upon request from the Centers for Disease Control and Prevention (CDC), the FDA issued an emergency use authorization (EUA) for IV peramivir while still an investigational antiviral drug, in hospitalized patients with H1N1pdm09 infection (Food and Drug Administration, 2015a). IV peramivir could only be used for hospitalized adult and pediatric patients who were not responsive to approved antivirals, or where delivery by alternative route (oral or inhaled) was not feasible. The EUA was effective through June 23, 2010. During this period, the CDC received 1371 requests for IV peramivir and 1274 hospitalized patients received ≥1 dose (median duration = 6 days; Yu et al., 2012). Under the EUA all AEs were to be reported to the FDA. Sorbello et al. (2012) reviewed 344 AE reports to evaluate the safety of IV peramivir treatment in hospitalized patients under the EUA. Many of the patients were severely ill requiring mechanical ventilation and/or renal replacement therapy. The most commonly reported AEs were death (18%), respiratory failure (8%), acute renal failure (7%), and acute respiratory distress syndrome (7%). But these AEs were not considered to be caused by IV peramivir and were thought to be a direct outcome of influenza infection induced illnesses. Death was mainly associated with other risk factors (i.e., obesity, immunosuppressed, or aged >65 years; Sorbello et al., 2012). Rash was the only AE attributed to peramivir and was reported in 13 cases. Hernandez et al. (2011) reviewed data for 31 adult (including pregnant women) and pediatric hospitalized patients with severe H1N1pdm09 viral pneumonia who received IV peramivir during the EUA (Hernandez et al., 2011). The study concluded that the drug was generally well tolerated and associated with complete recovery.

A survey of the public acceptance of peramivir use under the EUA revealed that 48% of the participants would probably or definitely take peramivir (Quinn et al., 2015). Seventy-nine percent indicated that they would definitely take it if recommended by their physician or no alternative treatments were available. Willingness to take peramivir increased with increased illness severity and was also affected by the individual’s trust in government actions (Quinn et al., 2015). Therefore, providing patients with relevant information on efficacy and safety of peramivir as well as potential risk of influenza complications is likely to improve patients’ acceptance of treatment.

## Conclusion

Peramivir is a highly potent inhibitor against influenza A and B viruses with a well-demonstrated safety profile. Administration via IV route provides an alternative for patients who cannot use oral drugs. Common side effects include gastrointestinal disorders and decreased neutrophil count and are self-limiting. While currently only approved for treatment of uncomplicated influenza in adults, clinical studies and post-marketing efficacy and safety data in pediatric populations provide promise for the potential of approval of peramivir in this highly susceptible population. The potential for emergence of resistant viruses and cross-resistance with oseltamivir remains a challenge for clinical practice. This highlights the need for continual development of antivirals targeting other influenza virus or host proteins while monitoring the effectiveness of existing drugs.

## Author Contributions

MA and HZ drafted the manuscript. EM helped summarizing literature for clinical studies and drug design. HZ edited the final draft.

## Conflict of Interest Statement

The authors declare that the research was conducted in the absence of any commercial or financial relationships that could be construed as a potential conflict of interest.
